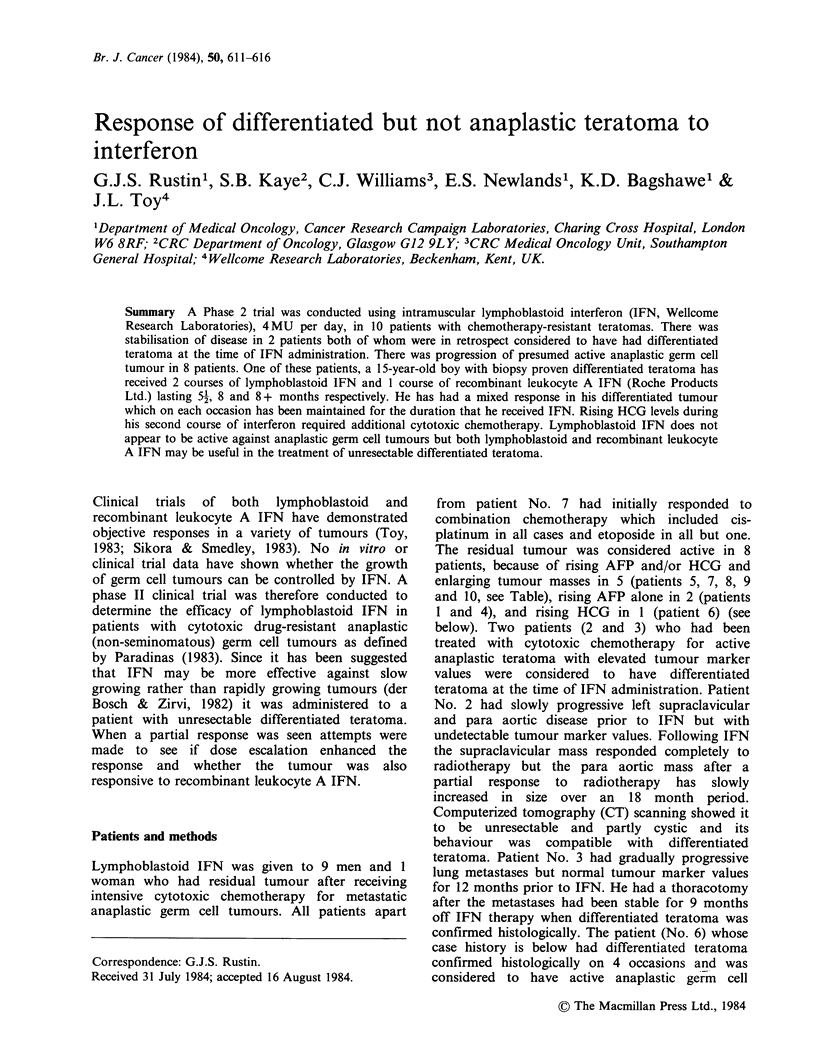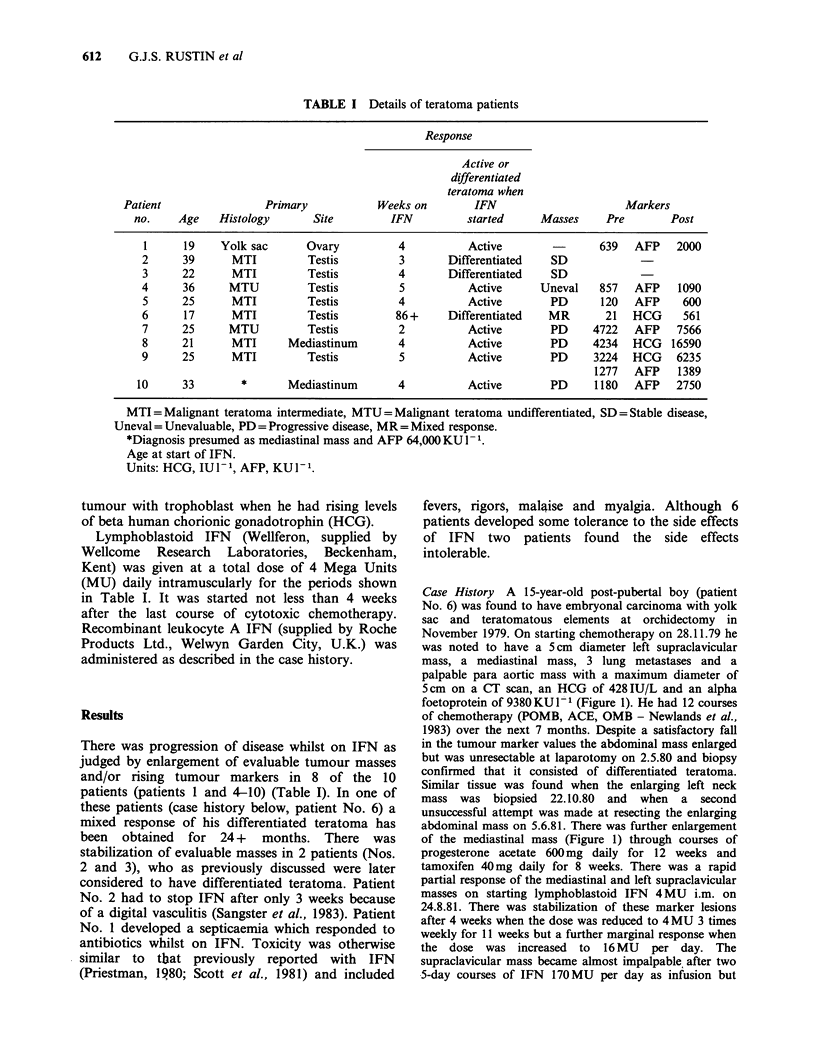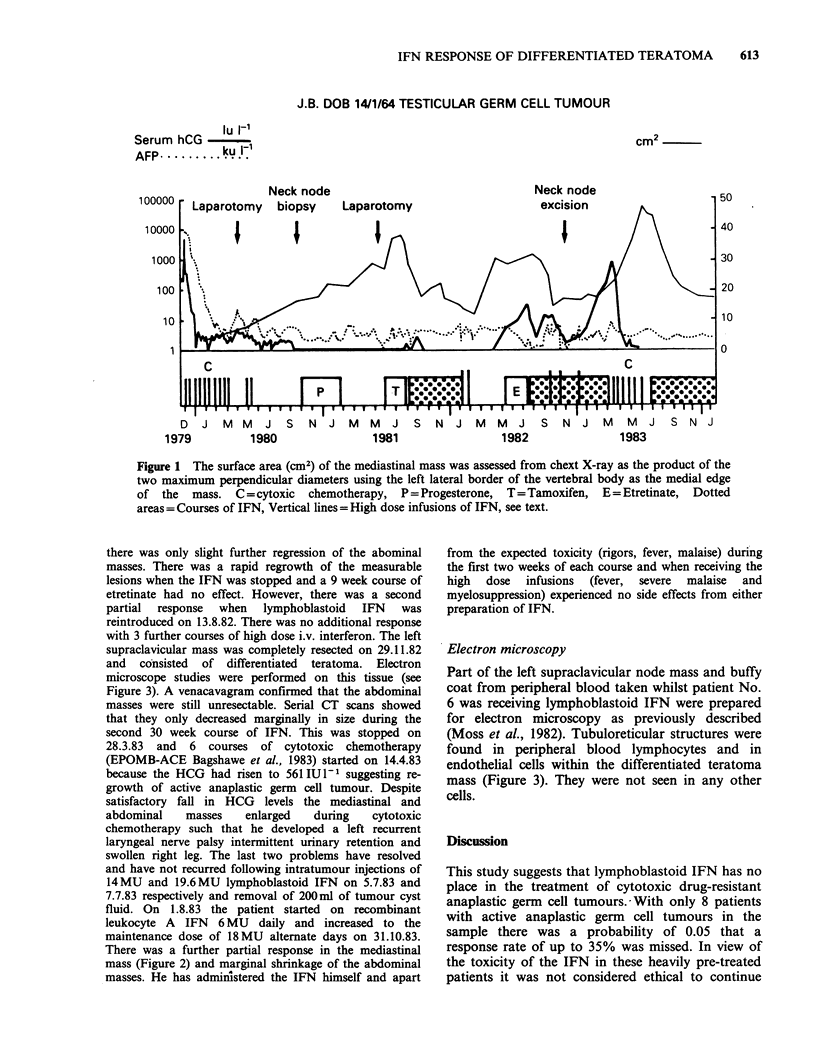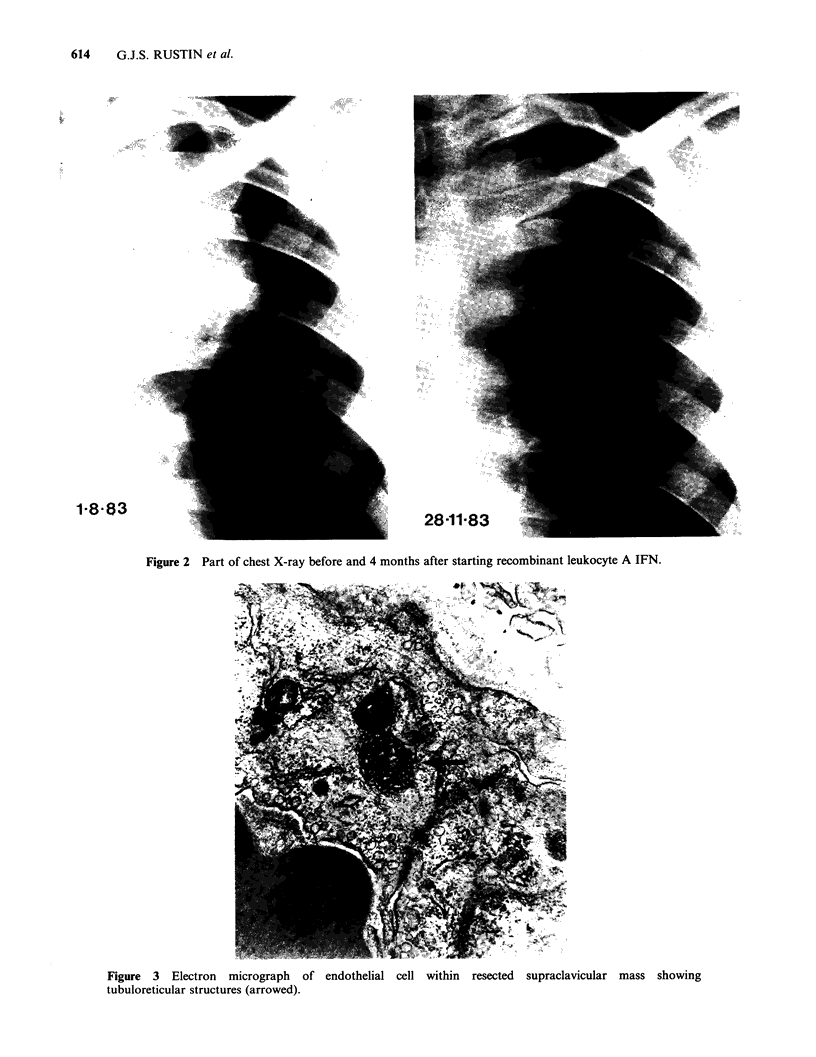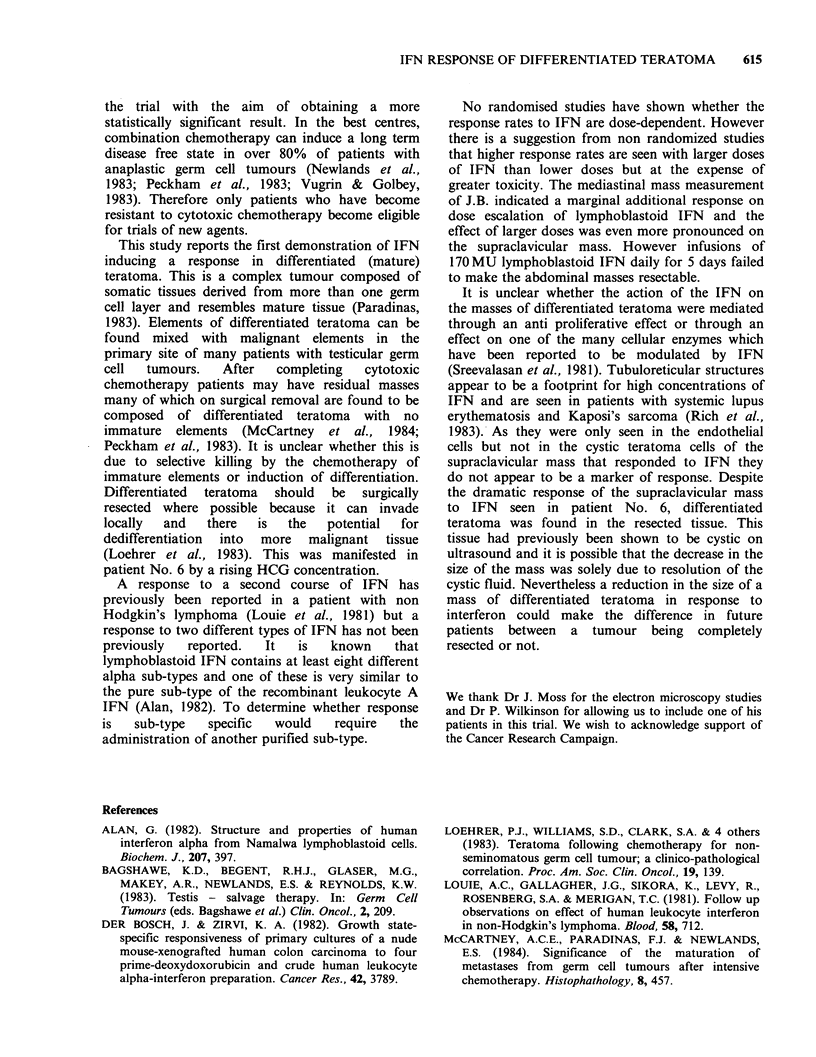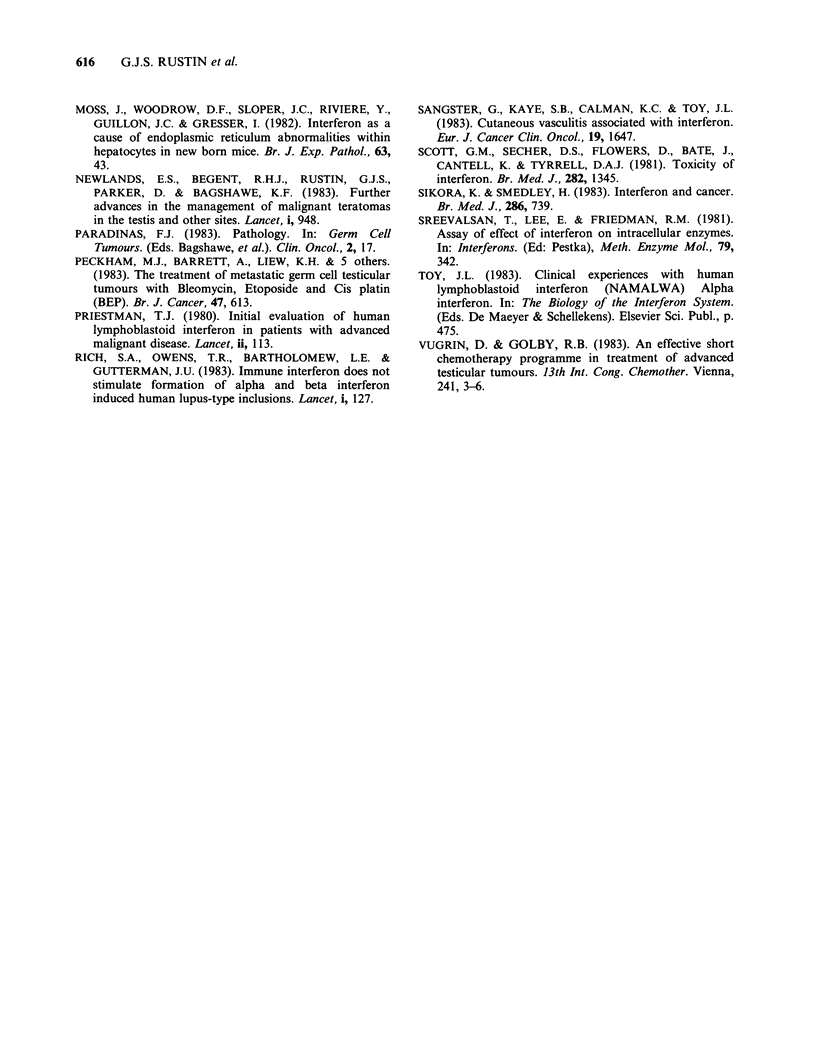# Response of differentiated but not anaplastic teratoma to interferon.

**DOI:** 10.1038/bjc.1984.227

**Published:** 1984-11

**Authors:** G. J. Rustin, S. B. Kaye, C. J. Williams, E. S. Newlands, K. D. Bagshawe, J. L. Toy

## Abstract

**Images:**


*Br. J. Cancer* (1984), **50**, 611–616

### Response of differentiated but not anaplastic teratoma to interferon

G.J.S. Rustin[1], S.B. Kaye[2], C.J. Williams[3], E.S. Newlands[1], K.D. Bagshawe[1] & J.L. Toy[4]

[1]*Department of Medical Oncology, Cancer Research Campaign Laboratories, Charing Cross Hospital, London W6 8RF;* [2]*CRC Department of Oncology, Glasgow G12 9LY;* [3]*CRC Medical Oncology Unit, Southampton General Hospital;* [4]*Wellcome Research Laboratories, Beckenham, Kent, UK.*

**Summary** A Phase 2 trial was conducted using intramuscular lymphoblastoid interferon (IFN, Wellcome Research Laboratories), 4 MU per day, in 10 patients with chemotherapy-resistant teratomas. There was stabilisation of disease in 2 patients both of whom were in retrospect considered to have had differentiated teratoma at the time of IFN administration. There was progression of presumed active anaplastic germ cell tumour in 8 patients. One of these patients, a 15-year-old boy with biopsy proven differentiated teratoma has received 2 courses of lymphoblastoid IFN and 1 course of recombinant leukocyte A IFN (Roche Products Ltd.) lasting $5\frac{1}{2}$, 8 and 8+ months respectively. He has had a mixed response in his differentiated tumour which on each occasion has been maintained for the duration that he received IFN. Rising HCG levels during his second course of interferon required additional cytotoxic chemotherapy. Lymphoblastoid IFN does not appear to be active against anaplastic germ cell tumours but both lymphoblastoid and recombinant leukocyte A IFN may be useful in the treatment of unresectable differentiated teratoma.

Clinical trials of both lymphoblastoid and recombinant leukocyte A IFN have demonstrated objective responses in a variety of tumours (Toy, 1983; Sikora & Smedley, 1983). No *in vitro* or clinical trial data have shown whether the growth of germ cell tumours can be controlled by IFN. A phase II clinical trial was therefore conducted to determine the efficacy of lymphoblastoid IFN in patients with cytotoxic drug-resistant anaplastic (non-seminomatous) germ cell tumours as defined by Paradinas (1983). Since it has been suggested that IFN may be more effective against slow growing rather than rapidly growing tumours (der Bosch & Zirvi, 1982) it was administered to a patient with unresectable differentiated teratoma. When a partial response was seen attempts were made to see if dose escalation enhanced the response and whether the tumour was also responsive to recombinant leukocyte A IFN.

### Patients and methods

Lymphoblastoid IFN was given to 9 men and 1 woman who had residual tumour after receiving intensive cytotoxic chemotherapy for metastatic anaplastic germ cell tumours. All patients apart from patient No. 7 had initially responded to combination chemotherapy which included cis-platinum in all cases and etoposide in all but one. The residual tumour was considered active in 8 patients, because of rising AFP and/or HCG and enlarging tumour masses in 5 (patients 5, 7, 8, 9 and 10, see Table), rising AFP alone in 2 (patients 1 and 4), and rising HCG in 1 (patient 6) (see below). Two patients (2 and 3) who had been treated with cytotoxic chemotherapy for active anaplastic teratoma with elevated tumour marker values were considered to have differentiated teratoma at the time of IFN administration. Patient No. 2 had slowly progressive left supraclavicular and para aortic disease prior to IFN but with undetectable tumour marker values. Following IFN the supraclavicular mass responded completely to radiotherapy but the para aortic mass after a partial response to radiotherapy has slowly increased in size over an 18 month period. Computerized tomography (CT) scanning showed it to be unresectable and partly cystic and its behaviour was compatible with differentiated teratoma. Patient No. 3 had gradually progressive lung metastases but normal tumour marker values for 12 months prior to IFN. He had a thoracotomy after the metastases had been stable for 9 months off IFN therapy when differentiated teratoma was confirmed histologically. The patient (No. 6) whose case history is below had differentiated teratoma confirmed histologically on 4 occasions and was considered to have active anaplastic germ cell

Correspondence: G.J.S. Rustin.
Received 31 July 1984; accepted 16 August 1984.

© The Macmillan Press Ltd., 1984

**TABLE I** Details of teratoma patients

| Patient no. | Age | Primary Histology | Primary Site | Response: Weeks on IFN | Response: Active or differentiated teratoma when IFN started | Masses | Markers Pre | | Markers Post |
|---|---|---|---|---|---|---|---|---|---|
| 1 | 19 | Yolk sac | Ovary | 4 | Active | — | 639 | AFP | 2000 |
| 2 | 39 | MTI | Testis | 3 | Differentiated | SD | | — | |
| 3 | 22 | MTI | Testis | 4 | Differentiated | SD | | — | |
| 4 | 36 | MTU | Testis | 5 | Active | Uneval | 857 | AFP | 1090 |
| 5 | 25 | MTI | Testis | 4 | Active | PD | 120 | AFP | 600 |
| 6 | 17 | MTI | Testis | 86+ | Differentiated | MR | 21 | HCG | 561 |
| 7 | 25 | MTU | Testis | 2 | Active | PD | 4722 | AFP | 7566 |
| 8 | 21 | MTI | Mediastinum | 4 | Active | PD | 4234 | HCG | 16590 |
| 9 | 25 | MTI | Testis | 5 | Active | PD | 3224 | HCG | 6235 |
| | | | | | | | 1277 | AFP | 1389 |
| 10 | 33 | * | Mediastinum | 4 | Active | PD | 1180 | AFP | 2750 |

MTI = Malignant teratoma intermediate, MTU = Malignant teratoma undifferentiated, SD = Stable disease, Uneval = Unevaluable, PD = Progressive disease, MR = Mixed response.
*Diagnosis presumed as mediastinal mass and AFP 64,000 $KU\,l^{-1}$.
Age at start of IFN.
Units: HCG, $IU\,l^{-1}$, AFP, $KU\,l^{-1}$.

tumour with trophoblast when he had rising levels of beta human chorionic gonadotrophin (HCG).

Lymphoblastoid IFN (Wellferon, supplied by Wellcome Research Laboratories, Beckenham, Kent) was given at a total dose of 4 Mega Units (MU) daily intramuscularly for the periods shown in Table I. It was started not less than 4 weeks after the last course of cytotoxic chemotherapy. Recombinant leukocyte A IFN (supplied by Roche Products Ltd., Welwyn Garden City, U.K.) was administered as described in the case history.

### Results

There was progression of disease whilst on IFN as judged by enlargement of evaluable tumour masses and/or rising tumour markers in 8 of the 10 patients (patients 1 and 4–10) (Table I). In one of these patients (case history below, patient No. 6) a mixed response of his differentiated teratoma has been obtained for 24+ months. There was stabilization of evaluable masses in 2 patients (Nos. 2 and 3), who as previously discussed were later considered to have differentiated teratoma. Patient No. 2 had to stop IFN after only 3 weeks because of a digital vasculitis (Sangster *et al.*, 1983). Patient No. 1 developed a septicaemia which responded to antibiotics whilst on IFN. Toxicity was otherwise similar to that previously reported with IFN (Priestman, 1980; Scott *et al.*, 1981) and included fevers, rigors, malaise and myalgia. Although 6 patients developed some tolerance to the side effects of IFN two patients found the side effects intolerable.

*Case History* A 15-year-old post-pubertal boy (patient No. 6) was found to have embryonal carcinoma with yolk sac and teratomatous elements at orchidectomy in November 1979. On starting chemotherapy on 28.11.79 he was noted to have a 5 cm diameter left supraclavicular mass, a mediastinal mass, 3 lung metastases and a palpable para aortic mass with a maximum diameter of 5 cm on a CT scan, an HCG of 428 IU/L and an alpha foetoprotein of 9380 $KU\,l^{-1}$ (Figure 1). He had 12 courses of chemotherapy (POMB, ACE, OMB – Newlands *et al.*, 1983) over the next 7 months. Despite a satisfactory fall in the tumour marker values the abdominal mass enlarged but was unresectable at laparotomy on 2.5.80 and biopsy confirmed that it consisted of differentiated teratoma. Similar tissue was found when the enlarging left neck mass was biopsied 22.10.80 and when a second unsuccessful attempt was made at resecting the enlarging abdominal mass on 5.6.81. There was further enlargement of the mediastinal mass (Figure 1) through courses of progesterone acetate 600 mg daily for 12 weeks and tamoxifen 40 mg daily for 8 weeks. There was a rapid partial response of the mediastinal and left supraclavicular masses on starting lymphoblastoid IFN 4 MU i.m. on 24.8.81. There was stabilization of these marker lesions after 4 weeks when the dose was reduced to 4 MU 3 times weekly for 11 weeks but a further marginal response when the dose was increased to 16 MU per day. The supraclavicular mass became almost impalpable after two 5-day courses of IFN 170 MU per day as infusion but

**Figure 1** The surface area (cm²) of the mediastinal mass was assessed from chext X-ray as the product of the two maximum perpendicular diameters using the left lateral border of the vertebral body as the medial edge of the mass. C = cytoxic chemotherapy, P = Progesterone, T = Tamoxifen, E = Etretinate, Dotted areas = Courses of IFN, Vertical lines = High dose infusions of IFN, see text.

there was only slight further regression of the abominal masses. There was a rapid regrowth of the measurable lesions when the IFN was stopped and a 9 week course of etretinate had no effect. However, there was a second partial response when lymphoblastoid IFN was reintroduced on 13.8.82. There was no additional response with 3 further courses of high dose i.v. interferon. The left supraclavicular mass was completely resected on 29.11.82 and consisted of differentiated teratoma. Electron microscope studies were performed on this tissue (see Figure 3). A venacavagram confirmed that the abdominal masses were still unresectable. Serial CT scans showed that they only decreased marginally in size during the second 30 week course of IFN. This was stopped on 28.3.83 and 6 courses of cytotoxic chemotherapy (EPOMB-ACE Bagshawe *et al.*, 1983) started on 14.4.83 because the HCG had risen to 561 IU $l^{-1}$ suggesting regrowth of active anaplastic germ cell tumour. Despite satisfactory fall in HCG levels the mediastinal and abdominal masses enlarged during cytotoxic chemotherapy such that he developed a left recurrent laryngeal nerve palsy intermittent urinary retention and swollen right leg. The last two problems have resolved and have not recurred following intratumour injections of 14 MU and 19.6 MU lymphoblastoid IFN on 5.7.83 and 7.7.83 respectively and removal of 200 ml of tumour cyst fluid. On 1.8.83 the patient started on recombinant leukocyte A IFN 6 MU daily and increased to the maintenance dose of 18 MU alternate days on 31.10.83. There was a further partial response in the mediastinal mass (Figure 2) and marginal shrinkage of the abdominal masses. He has administered the IFN himself and apart from the expected toxicity (rigors, fever, malaise) during the first two weeks of each course and when receiving the high dose infusions (fever, severe malaise and myelosuppression) experienced no side effects from either preparation of IFN.

### *Electron microscopy*

Part of the left supraclavicular node mass and buffy coat from peripheral blood taken whilst patient No. 6 was receiving lymphoblastoid IFN were prepared for electron microscopy as previously described (Moss *et al.*, 1982). Tubuloreticular structures were found in peripheral blood lymphocytes and in endothelial cells within the differentiated teratoma mass (Figure 3). They were not seen in any other cells.

### Discussion

This study suggests that lymphoblastoid IFN has no place in the treatment of cytotoxic drug-resistant anaplastic germ cell tumours. With only 8 patients with active anaplastic germ cell tumours in the sample there was a probability of 0.05 that a response rate of up to 35% was missed. In view of the toxicity of the IFN in these heavily pre-treated patients it was not considered ethical to continue

**Figure 2** Part of chest X-ray before and 4 months after starting recombinant leukocyte A IFN.

**Figure 3** Electron micrograph of endothelial cell within resected supraclavicular mass showing tubuloreticular structures (arrowed).

the trial with the aim of obtaining a more statistically significant result. In the best centres, combination chemotherapy can induce a long term disease free state in over 80% of patients with anaplastic germ cell tumours (Newlands *et al.*, 1983; Peckham *et al.*, 1983; Vugrin & Golbey, 1983). Therefore only patients who have become resistant to cytotoxic chemotherapy become eligible for trials of new agents.

This study reports the first demonstration of IFN inducing a response in differentiated (mature) teratoma. This is a complex tumour composed of somatic tissues derived from more than one germ cell layer and resembles mature tissue (Paradinas, 1983). Elements of differentiated teratoma can be found mixed with malignant elements in the primary site of many patients with testicular germ cell tumours. After completing cytotoxic chemotherapy patients may have residual masses many of which on surgical removal are found to be composed of differentiated teratoma with no immature elements (McCartney *et al.*, 1984; Peckham *et al.*, 1983). It is unclear whether this is due to selective killing by the chemotherapy of immature elements or induction of differentiation. Differentiated teratoma should be surgically resected where possible because it can invade locally and there is the potential for dedifferentiation into more malignant tissue (Loehrer *et al.*, 1983). This was manifested in patient No. 6 by a rising HCG concentration.

A response to a second course of IFN has previously been reported in a patient with non Hodgkin's lymphoma (Louie *et al.*, 1981) but a response to two different types of IFN has not been previously reported. It is known that lymphoblastoid IFN contains at least eight different alpha sub-types and one of these is very similar to the pure sub-type of the recombinant leukocyte A IFN (Alan, 1982). To determine whether response is sub-type specific would require the administration of another purified sub-type.

No randomised studies have shown whether the response rates to IFN are dose-dependent. However there is a suggestion from non randomized studies that higher response rates are seen with larger doses of IFN than lower doses but at the expense of greater toxicity. The mediastinal mass measurement of J.B. indicated a marginal additional response on dose escalation of lymphoblastoid IFN and the effect of larger doses was even more pronounced on the supraclavicular mass. However infusions of 170 MU lymphoblastoid IFN daily for 5 days failed to make the abdominal masses resectable.

It is unclear whether the action of the IFN on the masses of differentiated teratoma were mediated through an anti proliferative effect or through an effect on one of the many cellular enzymes which have been reported to be modulated by IFN (Sreevalasan *et al.*, 1981). Tubuloreticular structures appear to be a footprint for high concentrations of IFN and are seen in patients with systemic lupus erythematosis and Kaposi's sarcoma (Rich *et al.*, 1983). As they were only seen in the endothelial cells but not in the cystic teratoma cells of the supraclavicular mass that responded to IFN they do not appear to be a marker of response. Despite the dramatic response of the supraclavicular mass to IFN seen in patient No. 6, differentiated teratoma was found in the resected tissue. This tissue had previously been shown to be cystic on ultrasound and it is possible that the decrease in the size of the mass was solely due to resolution of the cystic fluid. Nevertheless a reduction in the size of a mass of differentiated teratoma in response to interferon could make the difference in future patients between a tumour being completely resected or not.

We thank Dr J. Moss for the electron microscopy studies and Dr P. Wilkinson for allowing us to include one of his patients in this trial. We wish to acknowledge support of the Cancer Research Campaign.

### References

ALAN, G. (1982). Structure and properties of human interferon alpha from Namalwa lymphoblastoid cells. *Biochem. J.*, **207**, 397.

BAGSHAWE, K.D., BEGENT, R.H.J., GLASER, M.G., MAKEY, A.R., NEWLANDS, E.S. & REYNOLDS, K.W. (1983). Testis – salvage therapy. In: *Germ Cell Tumours* (eds. Bagshawe *et al.*) *Clin. Oncol.*, **2**, 209.

DER BOSCH, J. & ZIRVI, K. A. (1982). Growth state-specific responsiveness of primary cultures of a nude mouse-xenografted human colon carcinoma to four prime-deoxydoxorubicin and crude human leukocyte alpha-interferon preparation. *Cancer Res.*, **42**, 3789.

LOEHRER, P.J., WILLIAMS, S.D., CLARK, S.A. & 4 others (1983). Teratoma following chemotherapy for non-seminomatous germ cell tumour; a clinico-pathological correlation. *Proc. Am. Soc. Clin. Oncol.*, **19**, 139.

LOUIE, A.C., GALLAGHER, J.G., SIKORA, K., LEVY, R., ROSENBERG, S.A. & MERIGAN, T.C. (1981). Follow up observations on effect of human leukocyte interferon in non-Hodgkin's lymphoma. *Blood*, **58**, 712.

McCARTNEY, A.C.E., PARADINAS, F.J. & NEWLANDS, E.S. (1984). Significance of the maturation of metastases from germ cell tumours after intensive chemotherapy. *Histophathology*, **8**, 457.

MOSS, J., WOODROW, D.F., SLOPER, J.C., RIVIERE, Y., GUILLON, J.C. & GRESSER, I. (1982). Interferon as a cause of endoplasmic reticulum abnormalities within hepatocytes in new born mice. *Br. J. Exp. Pathol.*, **63**, 43.

NEWLANDS, E.S., BEGENT, R.H.J., RUSTIN, G.J.S., PARKER, D. & BAGSHAWE, K.F. (1983). Further advances in the management of malignant teratomas in the testis and other sites. *Lancet*, **i**, 948.

PARADINAS, F.J. (1983). Pathology. In: *Germ Cell Tumours*. (Eds. Bagshawe, *et al.*). *Clin. Oncol.*, **2**, 17.

PECKHAM, M.J., BARRETT, A., LIEW, K.H. & 5 others. (1983). The treatment of metastatic germ cell testicular tumours with Bleomycin, Etoposide and Cis platin (BEP). *Br. J. Cancer*, **47**, 613.

PRIESTMAN, T.J. (1980). Initial evaluation of human lymphoblastoid interferon in patients with advanced malignant disease. *Lancet*, **ii**, 113.

RICH, S.A., OWENS, T.R., BARTHOLOMEW, L.E. & GUTTERMAN, J.U. (1983). Immune interferon does not stimulate formation of alpha and beta interferon induced human lupus-type inclusions. *Lancet*, **i**, 127.

SANGSTER, G., KAYE, S.B., CALMAN, K.C. & TOY, J.L. (1983). Cutaneous vasculitis associated with interferon. *Eur. J. Cancer Clin. Oncol.*, **19**, 1647.

SCOTT, G.M., SECHER, D.S., FLOWERS, D., BATE, J., CANTELL, K. & TYRRELL, D.A.J. (1981). Toxicity of interferon. *Br. Med. J.*, **282**, 1345.

SIKORA, K. & SMEDLEY, H. (1983). Interferon and cancer. *Br. Med. J.*, **286**, 739.

SREEVALSAN, T., LEE, E. & FRIEDMAN, R.M. (1981). Assay of effect of interferon on intracellular enzymes. In: *Interferons*. (Ed: Pestka), *Meth. Enzyme Mol.*, **79**, 342.

TOY, J.L. (1983). Clinical experiences with human lymphoblastoid interferon (NAMALWA) Alpha interferon. In: *The Biology of the Interferon System*. (Eds. De Maeyer & Schellekens). Elsevier Sci. Publ., p. 475.

VUGRIN, D. & GOLBY, R.B. (1983). An effective short chemotherapy programme in treatment of advanced testicular tumours. *13th Int. Cong. Chemother*. Vienna, 241, 3–6.